# KLK3 and TMPRSS2 for molecular lymph-node staging in prostate cancer patients undergoing radical prostatectomy

**DOI:** 10.1038/s41391-020-00283-3

**Published:** 2020-09-25

**Authors:** Lukas Lunger, Margitta Retz, Miriam Bandur, Marc Souchay, Elisabeth Vitzthum, Marion Jäger, Gregor Weirich, Tibor Schuster, Michael Autenrieth, Hubert Kübler, Tobias Maurer, Mark Thalgott, Kathleen Herkommer, Florestan Koll, Jürgen E. Gschwend, Roman Nawroth, Matthias M. Heck

**Affiliations:** 1 Department of Urology, Rechts der Isar Medical Center, Technical University of Munich, Munich, Germany; 2 Department of Pathology and Pathologic Anatomy, Rechts der Isar Medical Center, Technical University of Munich, Munich, Germany; 3 Department of Family Medicine, McGill University, Montreal, QC Canada; 4 Present Address: Department of Urology, University of Würzburg, Würzburg, Germany; 5 Present Address: Department of Urology and Martini-Klinik Prostate Cancer Center, University Hospital Hamburg-Eppendorf, Hamburg, Germany

**Keywords:** Diagnostic markers, Predictive markers

## Abstract

**Background:**

Lymph-node (LN) metastasis in prostate cancer (PC) is a main risk factor for tumor recurrence after radical prostatectomy (RP). Molecular analysis facilitates detection of small-volume LN metastases with higher sensitivity than histopathology. We aimed to prospectively evaluate six candidate gene markers for detection of pelvic LN metastases and to determine their ability to predict biochemical recurrence-free survival (bRFS) in patients treated with RP.

**Methods:**

The expression of kallikrein 2, 3, and 4 (KLK2, KLK3, and KLK4), prostate-specific membrane antigen (PSMA), transmembrane serine protease 2 (TMPRSS2) and transient receptor potential cation channel subfamily M member 8 (TRPM8) was assessed using qPCR. We analyzed LNs from 111 patients (intermediate PC, *n* = 32 (29%); high-risk PC, *n* = 79 (71%)) who underwent RP and extended pelvic lymph-node dissection without neoadjuvant treatment.

**Results:**

Overall, 2411 LNs were examined by molecular and histopathologic examination. Histopathology detected 69 LN metastases in 28 (25%) patients. KLK2 and KLK3 diagnostically performed best and classified all pN1-patients correctly as molecular node-positive (molN1/pN1). The concordance on LN level was best for KLK3 (96%). KLK2, KLK3, KLK4, PSMA, TMPRSS2, and TRPM8 reclassified 27 (24%), 32 (29%), 29 (26%), 8 (7%), 13 (12%), and 23 (21%) pN0-patients, respectively, as node-positive (pN0/molN1). On multivariable cox regression analysis molecular LN status (molN1 vs. molN0) using KLK3 (HR 4.0, *p* = 0.04) and TMPRSS2 (HR 5.1, *p* = 0.02) were independent predictors of bRFS. Median bRFS was shorter in patients with only molecular positive LNs (molN1/pN0) for KLK3 (24 months, *p* = 0.001) and for TMPRSS2 (12 months, *p* < 0.001) compared to patients with negative nodes (molN0/pN0) (median bRFS not reached).

**Conclusions:**

For diagnostic purposes, KLK3 showed highest concordance with histopathology for detection of LN metastases in PC patients undergoing RP. For prognostic purposes, KLK3 and TMPRSS2 expression were superior to histopathologic LN status and other transcripts tested for molecular LN status. We suggest a combined KLK3/TMPRSS2 panel as a valuable diagnostic and prognostic tool for molecular LN analysis.

## Introduction

Prostate cancer (PC) remains the most common cancer among men both in Europe and the US and the second to third leading cancer-specific cause of death [1]. Presence of lymph-node (LN) metastasis in patients with PC undergoing radical prostatectomy (RP) is the strongest risk factor for both, tumor recurrence and cancer-specific mortality [2, 3]. In patients with LN-positive PC, adjuvant treatment after RP with either androgen-deprivation therapy (ADT) alone or ADT in combination with pelvic radiation therapy (RT) has shown a benefit in overall survival (OS), cancer-specific survival (CSS), and progression-free survival (PFS) [4–7]. LN staging is crucial to assign node-positive patients to adjuvant treatment and thereby prolong CSS in these patients [8, 9].

Cancer recurrence occurs in about 20% of patients with postoperative node-negative status following RP and is even more frequently observed in patients with high-risk features [10–12]. This might be explained by LN-understaging due to insufficient extent of LN dissection or localization of LN metastases outside of the dissection template, leading to false node-negative results. Accordingly, an extended pelvic LN dissection (ePLND) respecting the lymphatic draining sites of the prostate including the obturatoric fossa, internal, external, and common iliac field has been recommended by the European Association of Urology guidelines [13]. Another reason might be the presence of small-volume metastases within the dissected tissue that remain undetected by standard histopathology.

Previous studies therefore aimed to address this issue by applying quantitative polymerase chain reaction (qPCR) for molecular LN analysis to enhance sensitivity in comparison with histopathology [14–16]. Our group recently established a novel, validated qPCR assay quantifying prostate-specific Kallikrein 3 expression (KLK3 gene coding for PSA (prostate-specific antigen)) in fresh-frozen tissue from LNs [17]. We applied this method in a prospective biomarker trial with intermediate and high-risk PC patients undergoing RP with ePLND [18]. Herein, we demonstrated upstaging of one third of patients as node-positive by molecular LN examination (molN1) despite histopathologic negative LN status (pN0). Moreover, molecular LN status was a better predictor for biochemical recurrence than histopathologic LN status. Thus, molecular LN analysis has the potential to guide adjuvant treatment as a diagnostic tool and eventually to improve survival in PC.

Despite advances in the field, studies investigating the most suitable marker for molecular LN-staging accounting for the genetic intratumor heterogeneity in PC remain scarce [19]. Several candidate genes have been described but common study limitations are arbitrary gene selection, retrospective trial design and a lack of direct comparison between the described genes [20, 21].

Following a literature review and subsequent preclinical evaluation of candidate genes we identified six genes (kallikrein 2, 3, and 4 (KLK2, KLK3, and KLK4), prostate-specific membrane antigen (PSMA), transmembrane serine protease 2 (TMPRSS2), and transient receptor potential cation channel subfamily M member 8 (TRPM8)) suitable for molecular LN analysis with high expression in PC and low expression in peripheral blood mononuclear cells (PBMCs), which can be used as a model for detection of disseminated tumor cells in LNs [22]. Subsequently, this study aimed to prospectively investigate molecular LN analysis comparing the six candidate genes with histopathologic LN analysis as part of the aforementioned prospective biomarker trial in patients with intermediate and high-risk PC undergoing RP with ePLND. Objectives of this study were the assessment of detection rate of each gene in comparison with standard histopathology for detection of LN metastases as well as the association with biochemical recurrence-free survival (bRFS).

## Patients and methods

The local ethics committee approved the present study (ID 2607/09), which was conducted according to the Declaration of Helsinki (NCT01615965). Written informed consent was obtained in all patients prior to participation. LN specimens were obtained from 111 prospectively enrolled patients who underwent open RP and ePLND between February 2010 and February 2013 for intermediate or high-risk PC at our institution according to D’Amico criteria (Gleason score at biopsy ≥ 7 or PSA ≥ 10 ng/ml or clinical tumor extension ≥ cT2b) [23]. The study was prospectively planned for the evaluation of the abovementioned six target genes. In a previously published, first analysis of this prospective biomarker trial we reported on the use of one of the six investigated candidate genes (KLK3) for molecular staging [18].

Exclusion criteria were the presence of any concomitant malignant tumor at the time of inclusion, previous radiation or ADT, acute or chronic infectious or inflammatory disease, severe cardiopulmonary, renal, hepatic or hematopoietic disease.

Open RP and ePLND were performed following a predefined template comprising the bilateral removal of LN tissue in the obturator fossa region and along the iliac vessels (external, internal, and common) [17].

The selection of target genes for this study, a detailed explanation on LN preparation and fixation, applied qPCR analyses, the method for cut-off determination as well as applied statistical methods are described in the Supplementary Material.

### Endpoints and follow-up

Follow-up for determination of PSA levels and postoperative PC treatment was obtained at 3 month intervals during the first postoperative year, every 6 months during the second postoperative year and once every 12 months thereafter. A confirmed postoperative PSA value of >0.2 ng/ml was considered as biochemical recurrence. bRFS was defined as the time from RP to biochemical recurrence.

Results are reported in compliance with the REMARK criteria [24]. Primary objectives of this study are the diagnostic accuracy of each gene to correctly identify node-positive patients also detected by standard histopathology (pN1), the ability of each gene to detect LN metastases in pN0-patients as well as the potential of each marker to yield prognostic information.

## Results

### Patient characteristics and histopathology

Patient characteristics have been reported previously [18] (Supplementary Table 1). In brief, median patient age was 67. The evaluation of preoperative PC cancer risk revealed that 32 (29%) patients had intermediate and 79 (71%) had high-risk PC according to D’Amico et al. criteria [23].

A total of 3173 LNs were harvested from 111 patients, corresponding to a median of 27 LNs/patient (range 9–78) (Supplementary Fig. 1). After retrieval, 2411 LNs had a diameter > 3 mm and were analysed by both histopathology and qPCR as described (median 21 LNs/patient, range 6–48).

Overall, histopathology detected 69 LN metastases in 28 (25%) patients. Of 69 LN metastases, 66 were detected in LNs >3 mm and were thus also available for molecular LN analysis. The remaining three LN metastases were identified in histopathology as micrometastases in LNs <3 mm. These LNs were found in patients that were already staged as node positive in LNs >3 mm.

### Comparison of detection rate of molecular and histopathologic lymph-node analysis

Detection rate of LN metastasis (patient- and lymph nodewise) according to molecular LN analysis and histopathology is given in Table 1.

**Table 1:** Patient and lymph-node stratification by molecular marker expression in lymph nodes.

		Molecular analysis		Molecular and histopathologic analysis			
Target	Stratification by	molN0	molN1	molN0/pN0	molN0/pN1	molN1/pN0	molN1/pN1
KLK2^a^	Pat. *n* (%)	56 (50.5)	55 (49.5)	56 (50.5)	0 (0)	27 (24.3)	28 (25.2)
	LN *n* (%)	2197 (91.1)	214 (8.9)	2190 (90.8)	7 (0.3)	152 (6.3)	62 (2.6)
KLK3^a^	Pat. *n* (%^b^)	51 (45.9)	60 (54.1)	51 (45.9)	0 (0)	32 (28.8)	28 (25.2)
	LN *n* (%^b^)	2122 (88.0)	289 (12.0)	2116 (87.8)	3 (0.1)	223 (9.2)	66 (2.7)
KLK4^a^	Pat. *n* (%)	54 (48.6)	57 (51.4)	52 (46.8)	2 (1.8)	31 (27.9)	26 (23.4)
	LN *n* (%)	2221 (92.1)	190 (7.9)	2210 (91.7)	11 (0.5)	132 (5.5)	58 (2.4)
PSMA^a^	Pat. *n* (%)	75 (67.6)	36 (32.4)	72 (64.9)	3 (2.7)	11 (9.9)	25 (22.5)
	LN *n* (%)	2316 (96.1)	95 (3.9)	2300 (95.4)	16 (0.7)	42 (1.7)	53 (2.2)
TMPRSS2^a^	Pat. *n* (%)	70 (63.1)	41 (36.9)	68 (61.3)	2 (1.8)	16 (14.4)	25 (22.5)
	LN *n* (%)	2296 (95.2)	115 (4.8)	2283 (94.7)	13 (0.5)	59 (2.4)	56 (2.3)
TRPM8^a^	Pat. *n* (%)	60 (54.1)	51 (45.9)	49 (44.1)	2 (1.8)	25 (22.5)	26 (23.4)
	LN *n* (%)	2269 (94.1)	142 (5.9)	2256 (93.6)	13 (0.5)	86 (3.6)	56 (2.3)

*KLK2, KLK3, KLK4* Kallikrein 2, 3, and 4, *PSMA* prostate-specific membrane antigen, *RP* radical prostatectomy, *TMPRSS2* transmembrane serine protease 2, *TRPM8* transient receptor potential cation channel subfamily M member 8.

^a^Threshold calculated based on a gamma distribution to identify 99% of true histopathologic negative LNs with a 99% level of confidence.

^b^Percentages refer to percent of total.

On patient level, both KLK3 and KLK2 identified all histopathological node-positive patients (*n* = 28; 100%) by molecular examination. KLK4 and TRPM8 were able to correctly identify 26/28 patients, respectively, whereas PSMA and TMPRSS2 identified a total of 25/28 patients node positive by standard histopathology. Compared to histopathology, molecular staging using KLK3, KLK2, KLK4, PSMA, TMPRSS2, and TRPM8 led to an upstaging from N0 to N1 of 32 (29%), 27 (24%), 31 (28%), 11 (10%), 16 (14%), and 25 (23%) patients, respectively, otherwise missed by standard histopathology (molN1/pN0).

On LN level KLK3 yielded the highest concordance with histopathologic positive LNs (66 of 69; 96%) followed by KLK2 (62 of 69; 90%), TMPRSS2 (56 of 69; 83%), KLK4 (56 of 69; 81%), PSMA (53 of 59; 77%), and TRPM8 (56 of 69; 81%). The concordance for different transcripts used for molecular LN analysis is depicted in Supplementary Fig. 2.

### Association of molecular and histopathologic lymph-node status with biochemical recurrence-free survival

Median follow-up for all 111 patients was 48 months (95% CI: 44–49 months). Overall, biochemical recurrence was observed in 52 patients (47%).

**Fig. 1:**
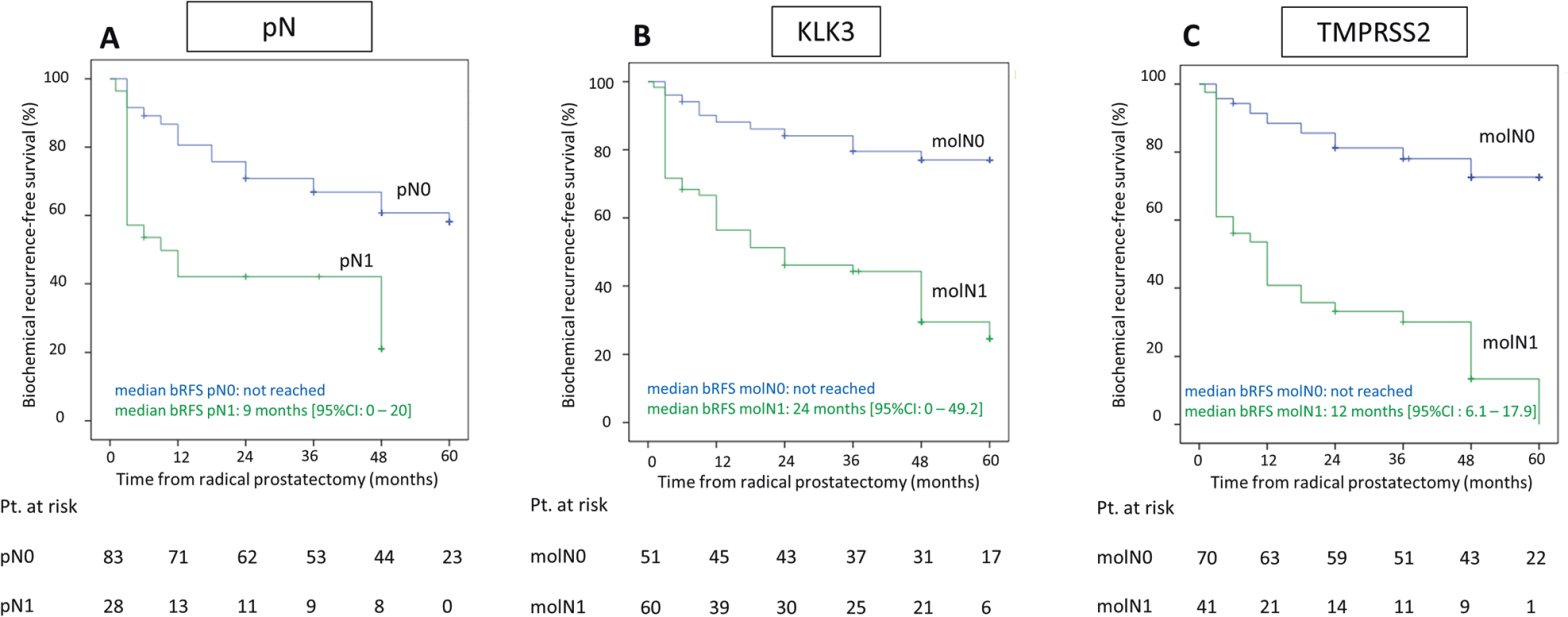
Kaplan–Meier curves for biochemical recurrence-free survival (bRFS) following radical prostatectomy according to histopathology or molecular lymph node analysis. Patients with positive lymph nodes, both by histopathology (**a**, pN1 vs. pN0) or molecular lymph-node analysis using KLK3 (**b**, molN1 vs. molN0), and TMPRSS2 (**c**, molN1 vs. molN0) had a shorter biochemical recurrence-free survival. Pt Patients.

In a multivariable cox regression model including established risk factors and LN status with dichotomous marker expression, TMPRSS2 (HR 5.1, 95% CI (1.4–19.0), *p* = 0.02) and KLK3 (HR 4.0, 95% CI (1.1–15.2), *p* = 0.04) remained the only statistically significant, independent predictors of bRFS and thus were superior to histopathologic LN examination (HR 1.1, 95% CI (0.5–2.2), *p* = 0.8) (Table 2a). Figure 1 shows the corresponding Kaplan–Meier curves for the estimation of bRFS by histopathology alone (pN0 vs. pN1), TMPRSS2 alone (molN1 vs. molN0) and KLK3 alone (molN1 vs. molN0), illustrating a shorter bRFS for patients with positive LNs. Median bRFS was 12 months (95% CI 6.1 vs. 17.9) in patients with TMPRSS2-positive LNs and not reached in patients in patients with TMPRSS2-negative LNs (log-rank *p* value < 0.001; Fig. 1b). Median bRFS was 24 months (95% CI 0 vs. 49.2) in patients with KLK3-positive LNs and not reached in patients in patients with KLK3-negative LNs (log-rank *p* value < 0.001; Fig. 1c).

**Table 2:** (A) Cox proportional multivariable regression analysis for the association of histopathologic lymph-node status (pN1 vs. pN0) with dichotomous molecular marker expression in lymph nodes (molN0 vs. molN1) and established risk factors with bRFS. (B) Cox proportional multivariable regression analysis for the association of histopathologic lymph-node status (pN1 vs. pN0), continuous molecular marker expression in lymph nodes and established risk factors with bRFS.

Variables	Category		*N*	Hazard ratio	95% CI	*p* value
**A**						
Lymph-node status	pN1 vs. pN0		28 vs. 83	1.1	0.5–2.2	0.8
	molN1 vs. molN0					
		KLK2	55 vs. 56	0.3	0.0–1.6	0.15
		KLK3	60 vs. 51	4.0	1.1–15.2	**0.04**
		KLK4	57 vs. 54	1.4	0.4–4.6	0.6
		PSMA	36 vs. 75	0.9	0.3–2.5	0.9
		TMPRSS2	41 vs. 70	5.1	1.4–19.0	**0.02**
		TRPM8	51 vs. 60	1.2	0.4–3.3	0.7
Preoperative PSA level	Continuous (per 10 ng/ml increase)		111	1.0	0.9–1.1	0.3
Tumor extension at RP	pT3 or pT4 vs. pT2		66 vs. 45	1.1	0.5–2.3	0.5
Gleason score at RP	8, 9 or 10 vs. 6 or 7		37 vs. 74	0.8	0.4–1.5	0.5
**B**						
Lymph-node status	pN1 vs. pN0		28 vs. 83	1.1	0.5–2.3	0.8
	Molecular continuous marker expression^a^					
		KLK2	111	1.4	0.8–2.5	0.3
		KLK3	111	1.1	0.7–2.0	0.6
		KLK4	111	0.8	0.4–1.7	0.5
		PSMA	111	0.6	0.3–1.2	0.16
		TMPRSS2	111	2.3	1.0–5.2	**0.04**
		TRPM8	111	0.7	0.5–1.1	0.13
Preoperative PSA level	Continuous (per 10 ng/ml increase)		111	1.0	0.9–1.1	0.8
Tumor extension at RP	pT3 or pT4 vs. pT2		66 vs. 45	0.8	0.4–1.7	0.5
Gleason score at RP	8, 9 or 10 vs. 6 or 7		37 vs. 74	1.0	0.6–1.9	0.9

Cox proportional multivariable regression analysis for the association of lymph-node status and established risk factors with bRFS. Lymph-node status is stratified according to histopathology as well as (A) dichotomous or (B) continuous molecular marker expression in lymph nodes.

*KLK2*, *KLK3*, *KLK4* Kallikrein 2, 3, and 4, *PSMA* prostate-specific membrane antigen, *RP* radical prostatectomy, *TMPRSS2* transmembrane serine protease 2, *TRPM8* transient receptor potential cation channel subfamily M member 8.

^a^Molecular continmarker expression = Log 10 (maximum marker expression in lymph nodes per patient + 1).

Bold values represent statistically significant results (*p* < 0.05).

In a multivariable cox regression model analysis including established risk factors and LN status with continuous molecular marker expression only TMPRSS2 remained an independent predictor of bRFS (Table 2b).

**Fig. 2:**
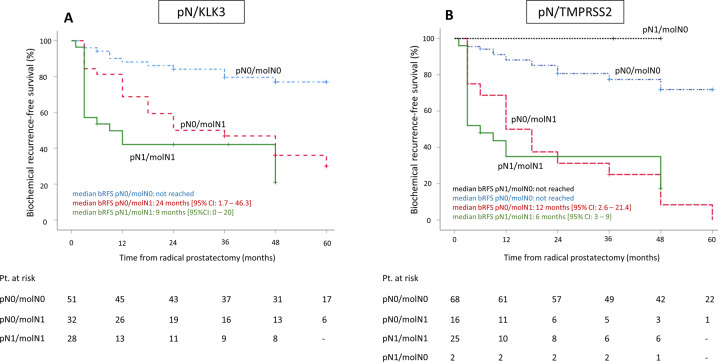
Kaplan–Meier curves for biochemical recurrence-free survival (bRFS) following radical prostatectomy according to the presence of lymph-node metastases detected by a combination of histopathology and molecular analysis. For both KLK3 (**a**) and TMPRSS2 (**b**), median bRFS was significantly shorter in molN1/pN1-patients and molN0/pN1-patients as compared to molN0/pN0-patients, in whom median bRFS was not reached. Pt Patients.

When combining both molecular and histopathologic LN status, we evaluated four patient risk groups with either negative LNs (molN0/pN0), only molecular positive LNs (molN1/pN0), only histopathologic positive LNs (molN0/pN1) or molecular and histopathologic positive LNs (molN1/pN1) and assessed their association with bRFS (Supplementary Table 2, Fig. 2). When compared to patients with LN-negative patients (molN0/pN0), both patients with only molecular positive LNs (molN1/pN0) as well as patients with molecular and histopathologic positive LNs exhibited a significantly elevated risk of biochemical recurrence for each of the selected genes (Supplementary Table 2). Figure 2 depicts the corresponding Kaplan–Meier curves for the combination of either histopathologic LN status with KLK3 (Fig. 2a) or histopathologic LN status with TMPRSS2 (Fig. 2b). For KLK3, median bRFS was significantly shorter in molN1/pN1-patients (9 months; (HR 5.4 (95% CI 2.6–11.5) *p* < 0.001) and molN0/pN1-patients (24 months; (HR 3.7 (95% CI 1.8–7.6) *p* = 0.001) compared to molN0/pN0-patients, in whom median bRFS was not reached. For TMPRSS2, median bRFS was significantly shorter in molN1/pN1-patients (6 months; (HR 5.4 (95% CI 2.8–10.5) *p* < 0.001) and molN0/pN1-patients (12 months; (HR 5.6 (95% CI 2.8–11.2) *p* < 0.001) compared to molN0/pN0-patients, in whom median bRFS also was not reached. Notably, two patients were LN-positive in histopathology but negative for TMPRSS2 expression (pN1/molN0) and both did not show biochemical recurrence during the observation period. One of the two pN1/molN0-patients received adjuvant ADT; the other patient, however, did not receive adjuvant radiation or ADT.

When combining molecular status for KLK3 and TMPRSS2, three risk groups were identified: patients negative for either marker (molN0_KLK3 and molN0_TMPRSS2), patients positive for KLK3 but negative for TMPRSS2 (molN1_KLK3 and molN0_TMPRSS2) as well as patients positive for both KLK3 and TMPRSS2 (molN1_KLK3 and molN1_TMPRSS2). There was no patient positive for TMPRSS2 but negative for KLK3. Positivity for both KLK3 and TMPRSS2 was a statistically significant predictor of biochemical recurrence-free survival (HR = 6.8 95% CI 3.4–13.5), while patients only positive for KLK3 showed an elevated risk, which was not statistically significant (HR = 1.7 95% CI 0.7–4.4) (Supplementary Fig. 3).

## Discussion

About 20% of patients develop biochemical recurrence after RP [25, 26]. The identification of patients at risk for biochemical recurrence is crucial to guide adjuvant treatment and thereby improve prognosis.

In a previous analysis of this prospective biomarker trial we demonstrated the diagnostic and prognostic superiority of molecular LN staging using expression of the prostate-specific gene KLK3 as compared to conventional histopathology for detection of LN metastasis in PC patients undergoing RP with ePLND for intermediate and high-risk PC [18]. Of note, there is a substantial genetic heterogeneity in PC and the optimal transcript used for molecular LN analysis has yet to be defined [19, 27].

In this study we prospectively compared the candidate transcripts KLK2, KLK3, KLK4, PSMA, TMPRSS2, and TRPM8 for molecular LN staging, which were identified from a systematic literature review and were selected based on systematic preclinical evaluation [22]. These transcripts were subsequently evaluated in a large, homogeneous cohort of intermediate and high-risk PC patients treated with RP and template ePLND. Our results suggest that for diagnostic purposes KLK2 or KLK3 quantification alone may suffice to identify patients harboring PC metastases. On patient level, each of both transcripts showed 100% concordance with histopathologic pN1-status and lead to upstaging from N0 to N1 in about one quarter of patients. In addition, on LN level KLK3 showed the highest concordance with histopathologic positive nodes and showed the highest sensitivity with upstaging from N0 to N1 suggesting that KLK3 analysis might be the optimal diagnostic transcript for molecular LN analysis.

Considering prognostic purposes, on the other hand, quantification of both KLK3 and TMPRSS2 in LNs were independent predictors of bRFS in a dichotomous marker expression model and TMPRSS2 was the only independent predictor in a continuous marker expression model. While TMPRSS2 missed two patients with histopathologic positive LNs, interestingly these patients did not develop biochemical recurrence. While in one patient this observation is explained by adjuvant ADT, the other patient did not receive any adjuvant treatment which provokes the hypothesis of lower risk of biochemical recurrence in patients with TMPRSS2-negative but histopathologic positive LNs. However, given the low prevalence this observation is hypothesis-generating at best and warrants further evaluation in larger patient cohorts. Taken together, our data support a combined approach of KLK3 and TMPRSS2 quantification in LNs to yield optimal diagnostic and prognostic information.

Our findings extend previous studies which proposed that a combination of either KLK3 with PSMA or KLK3 with KLK2 might most precisely identify PC metastases and predict biochemical recurrence [15, 28]. Miyake et al. prospectively evaluated the combination of KLK3 with PSMA for molecular LN staging and found that positivity for this combination was an independent predictor of bRFS [15, 16]. However, in their trial both transcripts were only analyzed as a combination and their unique utility for diagnostic and prognostic purposes was not reported. Kusuda et al. expanded the analysis of Miyake et al. by retrospectively quantifying the KLK2, PSCA, and DD3 in the same LNs obtained from 120 patients and compared them to KLK3 and PSMA-based molecular LN staging [16]. Kusuda et al. suggested that a combination of KLK3 and KLK2 most precisely predicted bRFS.

In our prospective trial, we only included candidate genes showing high expression in PC cell lines and low expression in PBMCs at preclinical evaluation [22]. PSCA and DD3 failed these criteria and were therefore not included. Nonetheless, the Kallikreins 2, 3 and 4 as well as PSMA, TMPRSS2 and TRPM8 passed these criteria. While our clinical trial validated the genes KLK2 and KLK3 as sufficient for diagnostic purposes to detect LN metastases, KLK3 and TMPRSS2 expression in LNs were identified as independent predictors of bRFS. A clinical application could be the use of KLK3 and TMPRSS2 to trigger adjuvant treatment such as radiation of the pelvic lymphatic draining sites, which has been shown to improve CSS in patients with limited histopathologic nodal involvement [4].

While the genes KLK2 and KLK3 have been studied extensively, only few reports exist on TMPRSS2. TMPRSS2, a member of the serine protease family, was found upregulated in PC and downregulated in androgen-independent PC, underlining its potential role in molecular PC detection. Ko et al. demonstrated that TMPRSS2 mediates maltriptase activation along with damage to the extracelluar matrix, hence promoting androgen-induced PC cell invasion, growth, and metastatic spread [29]. In PC, the 5-prime untranslated region of the TMPRSS2 gene was demonstrated to frequently bind to the oncogene ERG, generating TMPRSS2/ERG fusion transcripts [30]. One previous study confirmed the overexpression of TMPRSS2 mRNA in poorly differentiated PC [31]. Research on the expression of TMPRSS2 RNA expression in the human body revealed enhanced expression of TMPRSS2 RNA in the small intestine, pancreas and the prostate [32]. However, to our knowledge our study for the first time evaluated its use for molecular LN staging in PC.

A common limitation of molecular staging is that nodal morphology (e.g., metastasis size and extracapsular extension) cannot be obtained. However, mRNA transcript quantification by qPCR (especially considering small-volume metastases) shows increased sensitivity in comparison with standard histopathology and overcomes the issue of histopathology-associated sampling errors on nodal slicing or failure to detect small lesions on microscopic examination. Strengths of this study were above all its prospective design with inclusion of intermediate and high-risk PC patients treated with RP and a standardized template ePLND as well as the inclusion of candidate genes following systematic selection with preclinical evaluation and the determination of molecular cut-offs (molN1 vs. molN0) by a validated statistical method.

The results of this study suggest that the quantification of KLK3 by qPCR is sufficient for diagnostic purposes for molecular LN evaluation. For prognostic information, however, both KLK3 and TMPRSS2 were independent predictors for biochemical recurrence in PC patients undergoing RP with ePLND for intermediate and high-risk localized PC. A combined panel for KLK3 and TMPRSS2 transcript quantification ought to be evaluated prospectively to gain insight on stronger endpoints than bRFS.

## Supplementary information

Supplementary Methods, Tables and Figures

Supplementary Figure 1

Supplementary Figure 2

Supplementary Figure 3
