# Supplementary material for: KLK3 and TMPRSS2 for molecular lymph-node staging in prostate cancer patients undergoing radical prostatectomy
Source: Prostate Cancer Prostatic Dis. 2020 Sep 25;24(2):362–9. doi: 10.1038/s41391-020-00283-3 (PMC8134043; doi:10.1038/s41391-020-00283-3)
Supplement: Supplementary file 1 — Supplementary Methods, Tables and Figures [file 41391_2020_283_MOESM1_ESM.docx]

# Supplementary Methods

**Lymph node sampling and preparation**

Obtained LNs sized 3 to 20 mm were bisected perioperatively. Formalin fixation and subsequent hematoxylin and eosin staining (H&E) was performed in one half and the lateral edge of the second LN bisection for histopathologic staging (pN0 or pN1). The remaining tissue of the same LN was subsequently snap frozen within 30 minutes after removal and stored at -80°C for subsequent RNA extraction. For LNs > 20mm, bisections were created and examined like singular LNs < 20mm. In LNs < 3 mm, only histopathological examination was performed, as the tissue material did not suffice for further examination with either technique(1).

**Target gene selection**

The six genes of interest reported in this study were selected following a systematic literature review in 2013 and subsequent preclinical evaluation in PC cell lines and peripheral blood mononuclear cells (PBMCS) as described recently by our group (2). Briefly, the expression of 23 potential candidate genes with aberrant expression in PC cells or peripheral blood of PC patients was quantified in PC cell lines (LnCAP, VCAP) and compared to PBMCs obtained from five healthy male volunteers. A total of six genes (KLK3, KLK2, KLK4, PSMA, TMPRSS2 and TRPM8) with high expression in LnCAP or VCAP cells (Cq < 28) and absent or low expression in PBMCs (Cq > 38) were selected for further analysis.

**qPCR assay**

Molecular analysis of obtained LN specimen was performed using the validated qPCR assay established by our group(1).

LN tissue was homogenized and lysed for RNA extraction and subsequent complementary DNA (cDNA) generation. For detection of KLK3 (applied biosystems identification (ABI) Hs03063374_m1), KLK2 (ABI Hs00428383_m1), KLK4 (ABI Hs00191772_m1), PSMA (Hs00379515_m1), TMPRSS2 (ABI Hs00237175_m1) and TRPM8 (ABI Hs00375481_m1), commercially available Taqman probe and primer sets were used. Resulting reads were then normalized to endogenous reference gene expression of HPRT1 (ABI Hs01003267_m1) and UBC (ABI Hs00824723_m1) (Thermo Fisher – Applied Biosystems, Waltham, MA USA) in relation to a calibrator sample ((10 LnCAP cells spiked in 10^6^ peripheral blood mononuclear cells (PBMC) with a given relative gene expression of 1.0) using the ΔΔCT method.

**Determination of expression value cut-offs for candidate markers**

For determination of the physiologic KLK2, KLK3, KLK4, PSMA, TMPRSS2 and TRPM8 expression thresholds, qPCR analyses were performed in 143 LNs obtained from 25 male bladder cancer patients treated with cystoprostatectomy and histopathologic exclusion of PC as well as exclusion of bladder cancer metastasis (by qPCR for cytokeratin 20 expression and histopathologic examination). Gene expression cutoffs were calculated based on tolerance intervals accounting for a gamma distribution of all six genes of interest yielding 99% of true histopathologic negative LNs with a 99% level of confidence (3,4). According to these cutoffs (KLK2: 9.6; KLK3: 1.47; KLK4: 6.2; PSMA: 52; TMPRSS2: 280; TRPM8: 1.8), LNs were subsequently classified as molecular negative (molN0) or molecular positive (molN1).

**Statistical analysis:**

Data was analysed using IBM SPSS Statistics version 25.0 and the statistical software package R (5). Using the R package “tolerance” for estimating tolerance intervals based on a gamma distribution of all six genes of interest, 99% of true histopathologic negative LNs were classified with a 99% level of confidence among the investigated control-group (3,4). Standard descriptive statistics were performed to outline differences in node positive status (patient- and lymph nodewise) based on molecular and standard histopathology.

For estimation of sample size we preplanned to enrol at least 100 patients in order to account for the included molecular biomarkers together with further clinico-pathologic variables in multivariable regression models. Thus, the ratio between included patients and the number of included variables should be at least 10:1 (6).

Kaplan-Meier curves, log-rank statistics as well as uni- and multivariable Cox proportional hazard regression analyses were performed to investigate the association of marker positivity on bRFS. Hazard ratios (HR) and 95% confidence intervals (CI) were calculated. All statistical tests were performed two-sided, and a p value <0.05 was considered statistically significant.

**References:**

1. Heck MM, Retz M, Bandur M, Souchay M, Vitzthum E, Weirich G, et al. Topography of lymph node metastases in prostate cancer patients undergoing radical prostatectomy and extended lymphadenectomy: results of a combined molecular and histopathologic mapping study. Eur Urol [Internet]. 2014;66(2):222–9. Available from: http://www.ncbi.nlm.nih.gov/pubmed/23465520

2. Heck MM, Thalgott M, Schmid SC, Oh WK, Gong Y, Wang L, et al. A 2-Gene Panel Derived From Prostate Cancer-Enhanced Transcripts in Whole Blood Is Prognostic for Survival and Predicts Treatment Benefit in Metastatic Castration-Resistant Prostate Cancer. Prostate [Internet]. 2016;76(13):1160–8. Available from: http://www.ncbi.nlm.nih.gov/pubmed/27198487

3. Young DS. Tolerance: An R package for estimating tolerance intervals. J Stat Softw [Internet]. 2010 Aug 5 [cited 2020 Aug 1];36(5):1–39. Available from: https://www.jstatsoft.org/index.php/jss/article/view/v036i05/v36i05.pdf

4. Krishnamoorthy K, Mathew T, Mukherjee S. Normal-Based Methods for a Gamma Distribution: Prediction and Tolerance Intervals and Stress-Strength Reliability. 2008;

5. R-Core-Team. R: a language and environment for statistical computing [Internet]. 2018 [cited 2020 Aug 1]. Available from: https://www.gbif.org/tool/81287/r-a-language-and-environment-for-statistical-computing

6. Harrell F, Lee K, Mark D. Multivariable prognostic models: issues in developing models, evaluating assumptions and adequacy, and measuring and reducing errors. Stat Med [Internet]. 1996 [cited 2020 Jul 31];15(4). Available from: https://pubmed.ncbi.nlm.nih.gov/8668867/

# Supplementary Tables

***Legends:***

Supplementary Table 1: Pre-operative patient characteristics

Supplementary Table 2 Cox proportional univariable regression analysis for the association of dichotomous molecular marker expression in lymph nodes (molN0 vs. molN1) and histopathologic lymph node status with bRFS (pN1 vs pN0).

| Supplementary Table 1: Pre-operative patient characteristics | |
| --- | --- |
| **Age [years] (n=111)** |  |
| Mean (median) | 66 (67) |
| Interquartile range (range) | 61-71 (44-82) |
| No. patients (%) |  |
| < 65 | 43 (39) |
| 65-75 | 61 (55) |
| >75 | 7 (6) |
| **PSA [ng/ml] (n=111)** |  |
| Mean (median) | 22 (11) |
| Interquartile range (range) | 7-22 (1-367) |
| No. patients (%) |  |
| < 10 | 51 (46) |
| 10-20 | 31 (28) |
| > 20 | 29 (26) |
| **Gleason score at prostate biopsy (n=111)** |  |
| Mean (median) | 8 (8) |
| Interquartile range (range) | - 1. (6-10) |
| No. patients (%) |  |
| 6 | 8 (7) |
| 7 | 45 (41) |
| 8-10 | 58 (52) |
| **No. of cores taken at prostate biopsy (n=111)** |  |
| Mean (median) | 10 (10) |
| Interquartile range (range) | 10-12 (2-16) |
| **Prostate biopsy density, % (n=111)** |  |
| Mean (median) | 57 (50) |
| Interquartile range (range) | 33-80 (8-100) |
| **Clinical tumor stage, No. patients (%) (n=111)** |  |
| cT1c-2a | 52 (47) |
| cT2b-c | 57 (51) |
| cT3a-b | 2 (2) |
| **D´Amico risk group, No. patients (%) (n=111)** |  |
| Intermediate risk | 32 (29) |
| High risk | 79 (71) |

| Supplementary Table 2 Cox proportional univariable regression analysis for the association of dichotomous molecular marker expression in lymph nodes (molN0 vs. molN1) and histopathologic lymph node status with bRFS (pN1 vs pN0). | | | | | | | |
| --- | --- | --- | --- | --- | --- | --- | --- |
| ***Lymph node status*** | |  | ***N*** | ***Median bRFS***  ***(95% CI), months*** | ***HR*** | ***95% CI*** | ***P*** |
| KLK2 | pN0/molN1 vs. pN0/molN0 | | 27 vs. 56 | 24 (0.0-54.5) vs. NR | 3.3 | 1.6-6.7 | 0.001 |
|  | pN1/molN1 vs. pN0/molN0 | | 28 vs. 56 | 9 (0.0-20.1) vs. NR | 4.7 | 2.4-9.4 | <0.001 |
|  | pN1/molN0 vs. pN0/molN0 | | - | - | - | - | - |
| KLK3 | pN0/molN1 vs. pN0/molN0 | | 32 vs. 51 | 24 (1.7-46.3) vs. NR | 3.7 | 1.8-7.6 | 0.001 |
|  | pN1/molN1 vs. pN0/molN0 | | 28 vs. 51 | 9 (0.0-20.1) vs. NR | 5.4 | 2.6-11.5 | <0.001 |
|  | pN1/molN0 vs. pN0/molN0 | | - | - | - | - | - |
| KLK4 | pN0/molN1 vs. pN0/molN0 | | 31 vs. 52 | 36 (10.1-61.3) vs. NR | 3.3 | 1.6-6.8 | 0.001 |
|  | pN1/molN1 vs. pN0/molN0 | | 26 vs. 52 | 6 (2.4-9.6) vs. NR | 5.6 | 2.7-11.7 | <0.001 |
|  | pN1/molN0 vs. pN0/molN0 | | 2 vs. 52 | 48 (NA-NA) vs. NR | 1.8 | 0.2-14.2 | 0.6 |
| PSMA | pN0/molN1 vs. pN0/molN0 | | 11 vs. 72 | 24 (0.0-49.9) vs. NR | 3.7 | 1.8-7.9 | 0.001 |
|  | pN1/molN1 vs. pN0/molN0 | | 25 vs. 72 | 6 (3.0-9.0) vs. NR | 4.5 | 2.4-8.4 | <0.001 |
|  | pN1/molN0 vs. pN0/molN0 | | 3 vs. 72 | NR vs. NR | 0.9 | 0.1-6.7 | 0.9 |
| TMPRSS2 | pN0/molN1 vs. pN0/molN0 | | 16 vs. 68 | 12 (2.6-21.4) vs. NR | 5.6 | 2.8-11.2 | <0.001 |
|  | pN1/molN1 vs. pN0/molN0 | | 25 vs. 68 | 6 (3.0-9.0) vs. NR | 5.4 | 2.8-10.5 | <0.001 |
|  | pN1/molN0 vs. pN0/molN0 | | 2 vs. 68 | NR vs. NR | 0.0 | 0.0-NA | 1.0 |
| TRPM8 | pN0/molN1 vs. pN0/molN0 | | 25 vs. 58 | 24 (4.4-43.6) vs. NR | 3.1 | 1.5-6.2 | 0.002 |
|  | pN1/molN1 vs. pN0/molN0 | | 26 vs. 58 | 6 (2.4-9.6) vs. NR | 4.6 | 2.3-9.3 | <0.001 |
|  | pN1/molN0 vs. pN0/molN0 | | 2 vs. 58 | 48 (NA -NA) vs. NR | 3.2 | 0.7-14.2 | 0.12 |
| Abbreviations: bRFS = biochemical recurrence free survival; HR = hazard ratio; CI = confidence interval; KLK2, KLK3, KLK4 = Kallikrein 2, 3 and 4; NR = Not reached; PSMA = Prostate specific membrane antigen; RP= Radical prostatectomy; TMPRSS2 = Transmembrane serine protease 2; TRPM8 = transient receptor potential cation channel subfamily M member 8; | | | | | | | |

# Supplementary figures

***Legends:***

Supplementary Figure 1 Flow chart depicting the classification of lymph nodes and patients by histopathological examination (pN0 vs pN1). Three of 762 LNs (0.4%) sized < 3mm harbored additional micrometastases (pN1mi); however, these were present in 3 patients who were staged pN1/molN1 due to positive results in LNs ≥ 3mm. LNs=lymph nodes

Supplementary figure 2 Pie chart illustrating the total patient count (n=111) stratified by molecular marker expression in lymph nodes. Supplementary figure 2 depicts the concordance of different transcripts used for molecular LN analysis. A total of 69 (62%) patients were positive for at least one transcript (molN1) and in 33 (30%) patients all 6 transcripts were positive. Of note, all patients positive for KLK2, TMPRSS2 or PSMA were also positive for KLK3; thus, neither KLK2, nor TMPRSS2 or PSMA added diagnostic information. KLK3 identified 5 patients not positive for any other transcript. KLK4 identified 6 patients not positive for any other transcript and TRPM8 identified 1 patient not positive for any other transcript. All patients positive for TMPRSS2 were also positive for KLK3, amounting to a total of 41 patients with LNs positive for both KLK3 and TMPRSS2. A total of 19 patients were positive for KLK3 but negative for TMPRSS2. No patient was positive for TMPRSS2 but negative for KLK3.

Supplementary figure 3 Kaplan Meier curve for biochemical recurrence-free survival (bRFS) following radical prostatectomy according to the presence of lymph node metastases detected by molecular analysis for KLK3 and TMPRSS2. Pt = Patients. Only positivity for both markers was a significant predictor of bRFS (HR= 6.75, p<0.001).
