# Supplementary figures and images for: KLK3 and TMPRSS2 for molecular lymph-node staging in prostate cancer patients undergoing radical prostatectomy
Source: Prostate Cancer Prostatic Dis. 2020 Sep 25;24(2):362–9. doi: 10.1038/s41391-020-00283-3 (PMC8134043; doi:10.1038/s41391-020-00283-3)

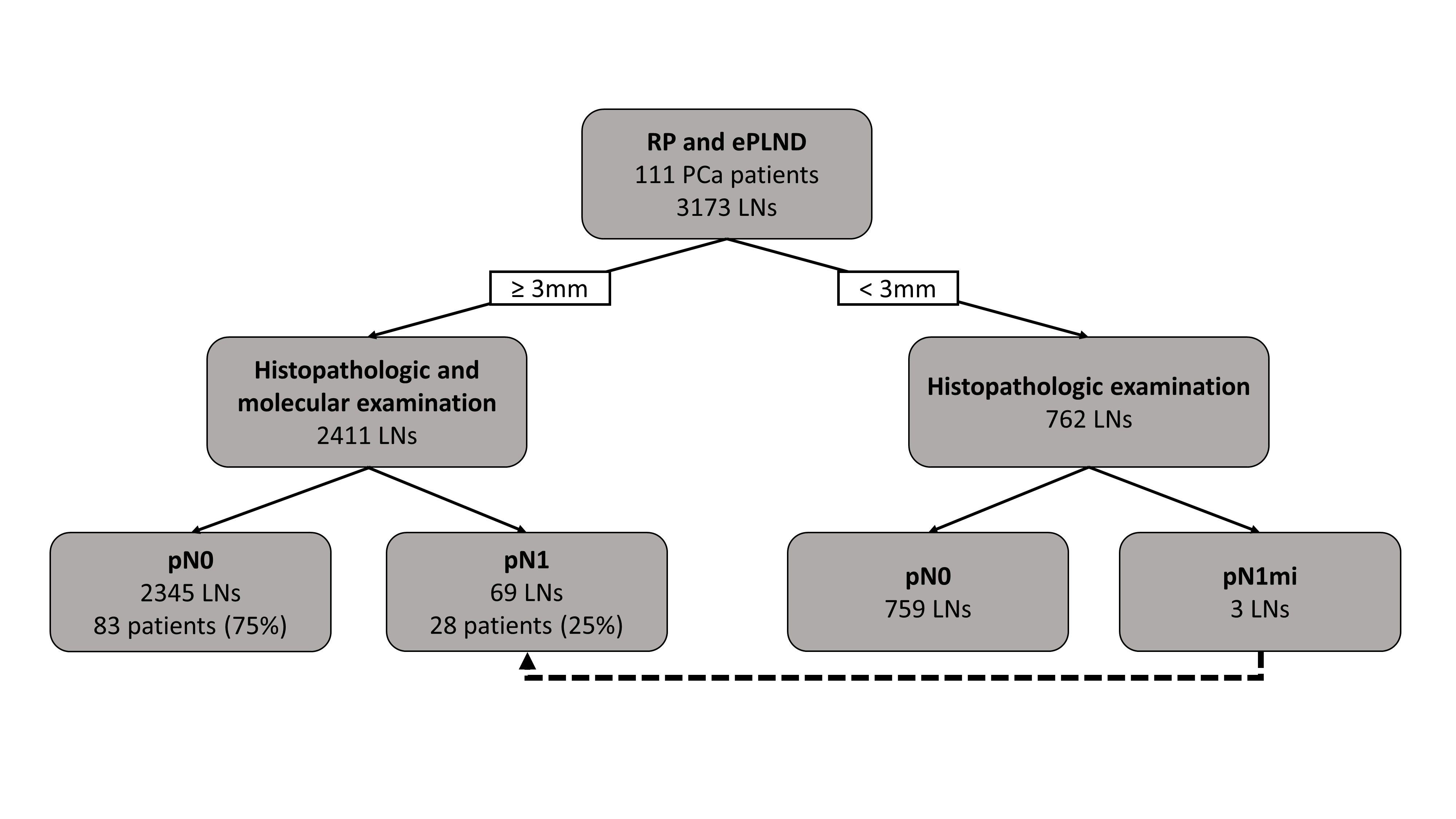

Supplement: Supplementary file 2 — Supplementary Figure 1 [file 41391_2020_283_MOESM2_ESM.tif]

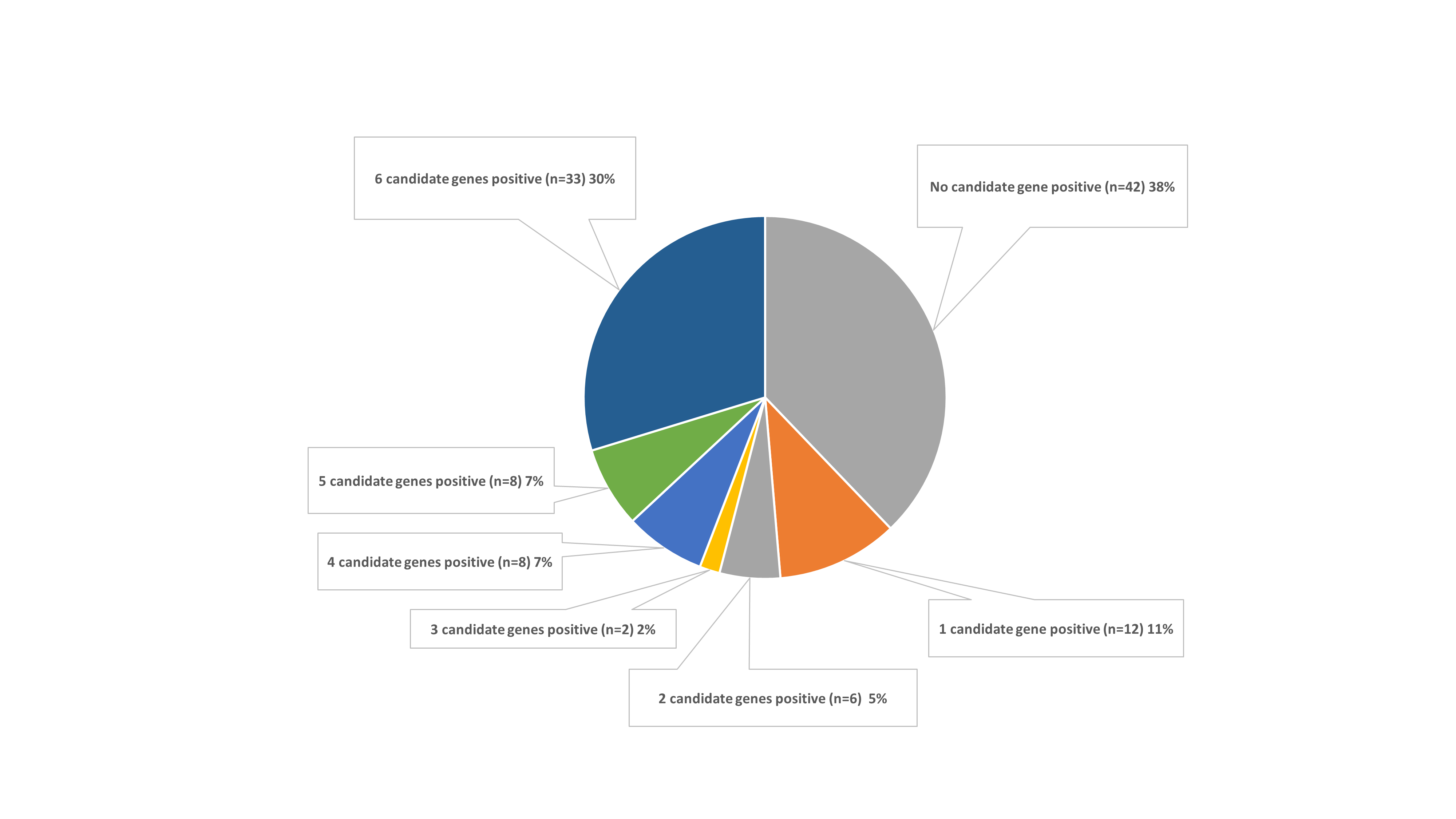

Supplement: Supplementary file 3 — Supplementary Figure 2 [file 41391_2020_283_MOESM3_ESM.tif]

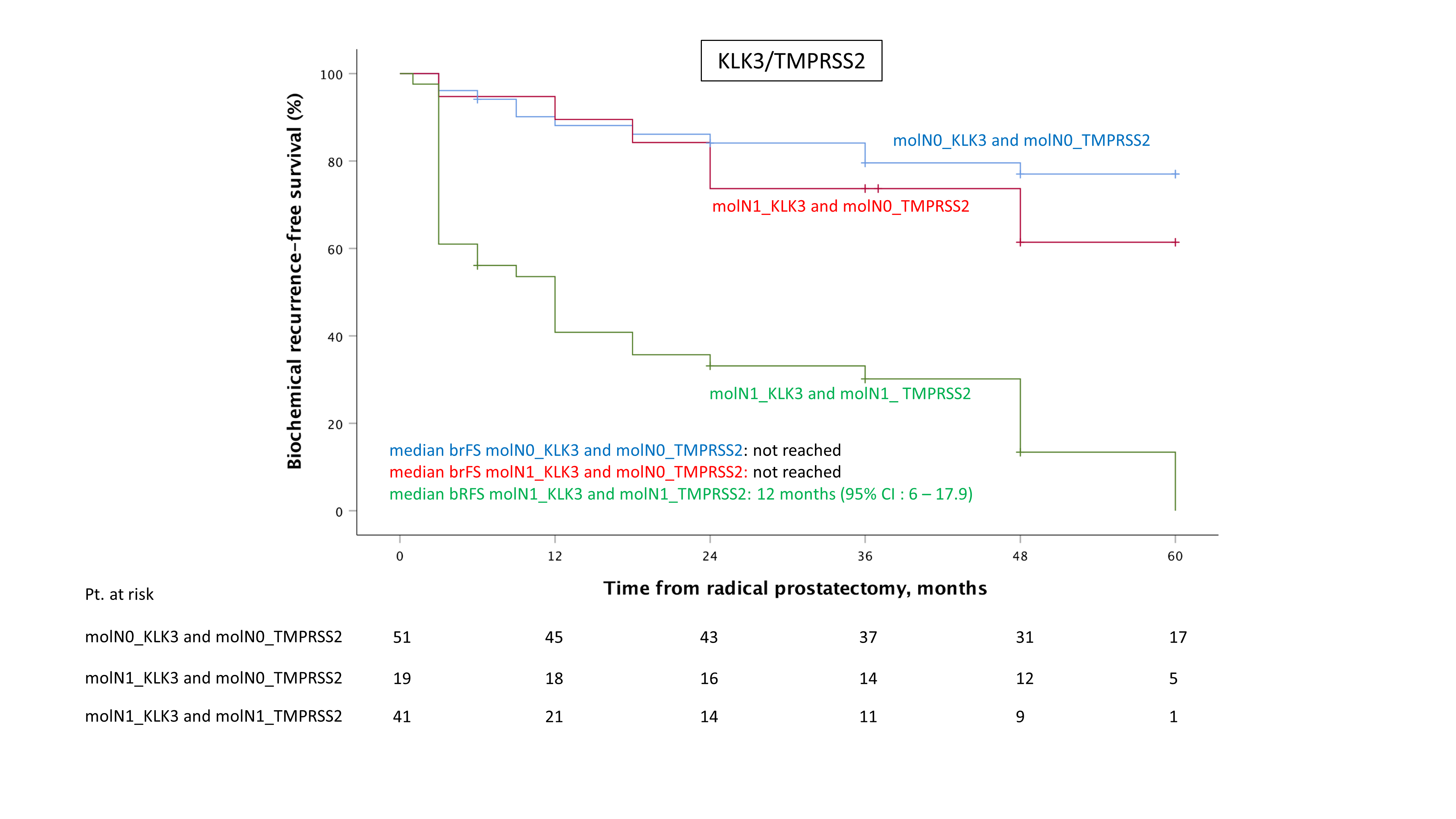

Supplement: Supplementary file 4 — Supplementary Figure 3 [file 41391_2020_283_MOESM4_ESM.tif]
